# Oral Gene Therapy of HFD-Obesity via Nonpathogenic Yeast Microcapsules Mediated shRNA Delivery

**DOI:** 10.3390/pharmaceutics13101536

**Published:** 2021-09-22

**Authors:** Li Zhang, Wan Zhang, Hang Peng, Yankun Li, Tongtong Leng, Chenxi Xie, Long Zhang

**Affiliations:** 1 The Second Affiliated Hospital of Xi’an Jiaotong University, Xi’an 710004, China; mli_zhang@outlook.com; 2 The Affiliated Zhongda Hospital of Southeast University, Nanjing 210009, China; 3 The First Affiliated Hospital of Xi’an Jiaotong University, Xi’an 710061, China; xjzhangw@stu.xjtu.edu.cn (W.Z.); ph1107980257@stu.xjtu.edu.cn (H.P.); leeky1229@stu.xjtu.edu.cn (Y.L.); 4 Frontier Institute of Science and Technology, Xi’an Jiaotong University, Xi’an 710054, China; lengtongtong@stu.xjtu.edu.cn (T.L.); chenxi-9509@stu.xjtu.edu.cn (C.X.)

**Keywords:** gene therapy, oral delivery, shRNA, yeast microcapsule

## Abstract

Obesity is a chronic systemic inflammatory disease, which occurs when energy intake exceeds the energy consumption. Therefore, controlling energy intake or increasing physical consumption can effectively control obesity. However, in reality, it is very difficult for the majority of obese patients to lose weight by autonomously controlling diet. In this study, oral shRNA/yeast microcapsules were constructed with non-virus-mediated *IL-1β* shRNA interference vectors and non-pathogenic *Saccharomyces cerevisiae*. Moreover, high-fat diet induced obese mice were established to assess the weight loss effect of *IL-1β* shRNA/yeast microcapsules via the oral route. After *IL-1β* shRNA/yeast treatment, body weight and fat weight was reduced. Compared with the control group, higher average food intake but lower energy conversion rate was observed in *IL-1β* shRNA/yeast group. In addition, lipid metabolism related cytokines and blood glucose concentration in the circulating blood was improved after *IL-1β* shRNA/yeast treatment. Yeast microcapsules mediated *IL-1β* shRNA delivery can effectively improve obesity. Noteworthy, this kind of non-diet-controlled weight loss strategy does not need diet control, and shows good biocompatibility. It is good news to obese patients who need to lose weight but cannot control their diet.

## 1. Introduction

Obesity seriously affects human life [1,2], and is closely linked to cardiovascular disease, type-2 diabetes, and atherosclerosis [3]. Obesity results from an imbalance of energy intake, which occurs when energy intake exceeds energy consumption [4]. Thus, controlling energy intake or increasing physical consumption can effectively control obesity. However, in reality, it is very difficult for the majority of obese patients to lose weight by autonomously controlling diet. Pharmacotherapy is an important approach to ameliorate patients’ life due to its ease of use and high patient compliance. Since some approved weight-loss drugs have a variety of side effects, such as vomiting, low efficiency and diarrhea [1], it is necessary to develop a safe and effective anti-obesity drug to improve the living conditions of patients and prevent the development of obesity into other related diseases.

Studies have shown that *IL-1β* is related to many chronic diseases such as rheumatoid arthritis, obesity, and type II diabetes [5]. It is regarded as a new type fat secretory adipokine with insulin resistance. Obesity is systemic inflammation, which can induce macrophages to produce IL-1β and increase the circulating IL-1β concentration. Blocking *IL-1β* with IL-1 receptor antagonist (IL-1Ra) could ameliorate blood glucose and β-cell function in obese mice [6,7]. The regulation of IL-1β expression by RNA interference could be an effective approach for the treatment of high-fat induced obesity, since gene therapy is a potential way for disease treatment and tissue function recovery [8,9]. How to deliver therapeutic genes such as miRNA or shRNA has become an important challenge of gene therapy in vivo.

*Saccharomyces cerevisiae* is widely used in wine-making and bread baking [10]. Moreover, yeast can effectively resist the degradation of gastro-intestinal tract and be used for nano-materials, miRNA, or protein delivery by oral administration [11,12,13,14,15]. As one of the main components of the yeast wall, β-glucan could be specifically recognized by dectin-1 receptor on the surface of macrophage [14,15]. Studies have shown that yeast can be engulfed by intestinal macrophages after oral administration. The phagocytosed yeast can be accompanied when macrophages migrate from the gut to other tissues [14,15,16,17]. Finally, yeast-macrophages system participates in immune regulation or gene therapy in inflammatory tissues [12,18]. We have previously proved that yeast-mediated oral miRNA or shRNA delivery can modulate macrophage related inflammatory response in the treatment of osteoarthritis [18,19].

**Scheme 1:**
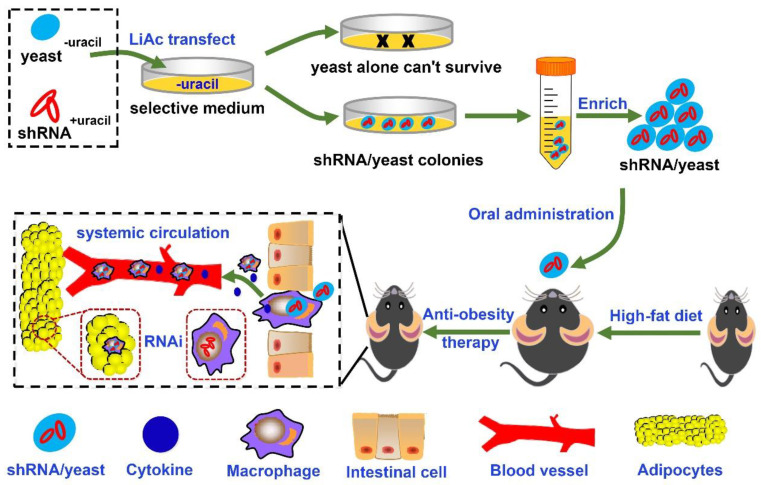
Schematic illustration of anti-obesity therapy by yeast microcapsules mediated oral delivery of shRNA.

Therefore, we hypothesized that yeast microcapsules as *IL-1β* shRNA delivery vehicle has potential application value in the treatment of obesity via the oral route. The purpose of the this study is to develop an efficient and safe shRNA delivery system for the treatment of chronic diseases, not limited to diet-induced obesity therapy [20]. The principle of this study is illustrated in Scheme 1.

## 2. Materials and Methods

### 2.1. Construction of IL-1β shRNA Vectors

ShRNA sequences of *IL-1β* shRNA were designed according to the method we previously described [11]. Then shRNA sequences were synthesized by GeneCreate (Wuhan, China) and inserted into the yeast cloning vector pIN-hU6 to get shRNA plasmids. EcoRI and XhoI restriction cloning site was added to the 5′ and 3′ ends of shRNAs respectively. Four *IL-1β* shRNAs (control shRNA, shRNA1, shRNA2, shRNA3) were cloned into pin27-hu6 vector by double digestion and ligation to construct pin27-hu6-shRNA interference vectors.

### 2.2. Adipocyte Culture

Pre-adipocytes 3T3-L1 were cultured in high glucose Dulbecco’s modified Eagle’s medium (HyClone) with 10% FBS and 1% penicillin streptomycin. Cell differentiation was induced by adding cell culture medium I (DMEM with 10% FBS, 1% P/S, 0.5 mM 3-isobutyl-1-methylxanthine (I7018, Sigma, Shanghai, China), 1 µM dexamethasone (D4902, Sigma, Shanghai, China) and 10 µg/mL insulin (I9278, Sigma, Shanghai, China)) for two days. Then, they continued to culture in cell culture medium II (DMEM with 10% FBS, 1% P/S and 10 µg/mL insulin) for another two days. Then cell culture medium (DMEM with 10% FBS and 1% P/S) was changed every two days for two times according to the method we described earlier [21].

### 2.3. Function of IL-1β shRNA in Lipid Metabolism

Lipo/Ctrl shRNA (Lipofectamine3000 with control shRNA) and Lipo/*IL-1β* shRNA (lipo3000 with *IL-1β* shRNA) were transfected into adipocytes. Related genes expression was detected by RT-qPCR method. And lipid droplets was detected by Oil-Red O Staining.

### 2.4. Preparation of IL-1β shRNA/Yeast Microcapsules

Three *IL-1β* shRNA/yeast and one control shRNA/yeast was constructed according to the method we previously described [11,18]. The *Saccharomyces cerevisiae* Scy27 preserved in our laboratory was used in this study. Three *IL-1β* shRNA plasmids pIN27-*IL-1β*-shRNA and one control shRNA plasmid pIN27-shRNA (negative control) were respectively transformed into Scy27 and cultured in the yeast incubator at 30 °C for 3 days to construct shRNA/yeast microcapsules [11]. Since yeast^−uracil^ cannot express uracil by itself, only shRNA/yeast microcapsules that contain shRNA^+uracil^ can survive in the selection medium lacking uracil. Single yeast colony was cultured in the uracil deficient liquid medium. After identification by PCR and sequencing, the control shRNA/yeast and *IL-1β* shRNA/yeasts were amplified.

### 2.5. Function Detect of shRNA/Yeast in Macrophage

RAW264.7 was used to prove shRNA/yeast microcapsule can be engulfed by macrophages. RAW264.7 was cultured in DMEM (ATCC, 30-2002, Manassas, VA, USA) containing 10% FBS and 100 ng/mL lipopolysaccharide (Sigma, L2880, Shanghai, China) for 24 h. Then 10^5^/6-well *IL-1β* shRNA/yeast microcapsule (three different *IL-1β* shRNA/yeast were mixed 1:1:1) and control shRNA/yeast was separately added into the lipopolysaccharide induced macrophages. After incubated for 4 h, a large number of *IL-1β* shRNA/yeast microcapsules were engulfed by macrophages. After 24 h treatment, the gene expression of *IL-1β* and *TNF-α* was quantified by RT-qPCR (Primers were showed in Table 1).

### 2.6. Mice and Diets

C57BL/6 male mice aged eight weeks were purchased from the Animal Breeding and Research Centre of Xi’an Jiaotong University, China. All mice were treated according to the policy of the Institutional Animal Care and Use Committee with room temperature and dark-light cycles (No.:2021-1493, 9 January 2021). After one week of normal chow diet acclimatization, the mice were fed with high-fat diet (Research Diets D12492, New Brunswick, NJ, USA) for 50 days to make obesity model mice. Then the obese mice were randomly allocated into three groups, NCD (normal diet with PBS, *n* = 9), HFD-Ctrl (high fat diet with control shRNA/yeast, *n* = 9) and HFD-Exp (high fat diet with *IL1β* shRNA/yeast, *n* = 11) on day 1. Mice were orally administered with 10 mg/kg shRNA/yeast every 2 days from day 1 to day 29. The body weight, food intake and blood glucose was detected and recorded every week from day 1. Glucose tolerance test was measured on day 28. Serum samples and major organs such as lung, liver, abdominal fat and spleen were collected on day 29.

### 2.7. Glucose Tolerance Test

After oral gavage of *IL1β* shRNA/yeast for 28 days, mice were randomly selected to fast overnight. And the blood glucose levels were measured (Roche Diagnostics, Basel, Switzerland) at 0, 15, 30, 60, and 120 min after intraperitoneal injection of 2 g/kg glucose.

### 2.8. Small Intestinal Macrophage Isolation

Small intestinal macrophages were isolated using the method we previously described [19]. Briefly, after cutting the small intestine into 1–2 mm^3^ size pieces, 25 mL of 1 mg/mL collagenase IV solution was added. Then the samples were digested at 37 °C, 155 rpm for 60 min. The digested cells were re-suspended with cells buffer (PBS with 0.5% BSA, 2 mM EDTA). According to the manufacturer’s instruction, CD11b microBeads (130-049-601, MiltenyiBiotec, Shanghai, China) were added into the cells suspension for macrophages sorting.

### 2.9. RT-qPCR and ELISA Assay

The mice blood samples were collected for enzyme linked immunosorbent assay. The processed serum was used for IL-1β (MLB00C, RD, Shanghai, China), adiponectin (MRP300, RD, Shanghai, China), insulin (80-INSMSU-E01, Alpco, Salem, MA, USA) and leptin (MOB00, RD, Shanghai, China) detection according to the manufacturers’ instructions. Total RNA was extracted from cells or tissue samples by using RNAiso Plus (9109, TaKaRa, Beijing, China) accordance with the manufacturer’s instructions. And the cDNA was synthesized using the First-Strand cDNA Synthesis Kit (G486, ABM, Beijing, China).

### 2.10. Histological Staining

Mice tissues such as spleen, abdominal fat, lung and liver were collected for histological staining. The tissue samples were fixed overnight in 4% paraformaldehyde, then dehydrated and embedded in paraffin for H&E staining. The liver was stained with oil red O and the small intestine was stained with immunofluorescence. Mice knee joints were collected for immunohistochemistry and Safranin O/Fast Green staining.

### 2.11. Statistical Analysi

All statistical results were presented as means ± SD. Unpaired two-tailed Student’s *t*-test was used for comparisons between two groups. A *p* value of <0.05 was considered statistically significant. Results were analyzed using Prism version 8 (GraphPad Software Inc., San Diego, CA, USA) for Windows.

## 3. Results

### 3.1. IL-1β shRNA Knock Down IL-1β Expression In Vitro

According to mouse *IL-1β* mRNA sequence, we designed three different shRNA targeted sequences based on bioinformatic analysis (Figure S1A and Table S1). The shRNA vector contained miR30 flanking sequence which could enhance the shRNA expression [11]. To achieve the maximum inhibition efficiency, we mixed three shRNAs in equal proportion (1:1:1). The shRNA vectors and the reporter vector pRP-CMV-IL1β-GFP (Figure S1B) were co-transfected into 293T cells. Compared to the control groups (negative control group and Ctrl shRNA group), the green fluorescence intensity was knocked down in *IL-1β* shRNA group (Figure S1C). Fluorescence intensity was quantified by image J (Figure S1D). This suggested that *IL-1β* shRNA could down regulate the expression of *IL-1β* in mammalian cells.

### 3.2. IL-1β shRNA Increased Lipid Metabolism in Adipocytes

Figure S2 showed the effect of *IL-1β* shRNA on lipid and obesity related genes expression in adipocytes. Compared with the control groups, lipid expression in Lipo/*IL-1β* shRNA group was down regulated (Figure S2A). And the percentage of oil drop area in different groups was quantified (Figure S2B). Compared with the control groups, the expression of obesity related gene *IL-1β* (Figure S2C) and FAS (Figure S2D) was decreased in the experimental group. These results indicated that *IL-1β* shRNA has the ability to inhibit lipid expression in adipocytes.

### 3.3. Construction of IL-1β shRNA/Yeast Microcapsule and Its Functional Verification on Macrophage

**Figure 1:**
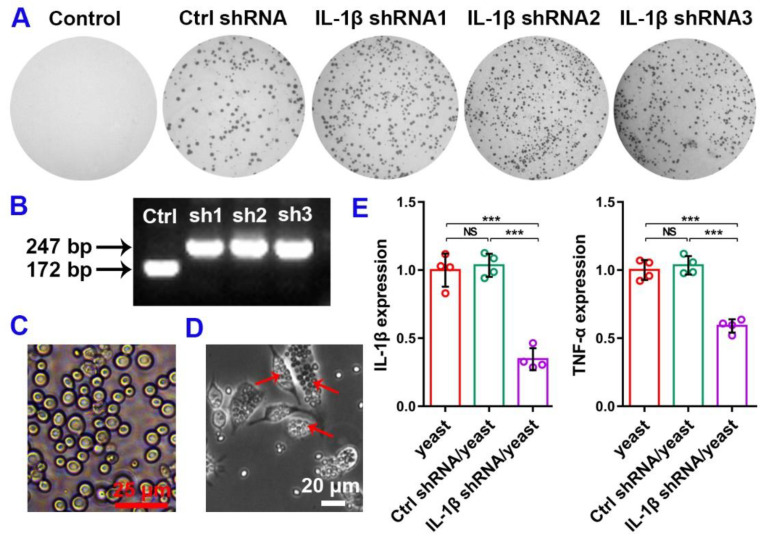
Construction and functional verification of shRNA/yeast. (**A**) Construction of shRNA/yeast by chemical transformation of shRNA plasmid. Only the shRNA/yeast containing shRNA^+uracil^ can survive in uracil deficient medium. (**B**) PCR detection of single yeast clone (Ctrl, sh1, sh2, sh3 refer to control shRNA/yeast, *IL-1β* shRNA1/yeast, *IL-1β* shRNA2/yeast and *IL-1β* shRNA3/yeast). (**C**) Morphology of shRNA/yeast in bright field. (**D**) shRNA/yeast was engulfed by macrophage. (**E**) *IL-1β* and *TNF-α* expression in macrophages after treatment with *IL-1β* shRNA/yeast. NS (no significance). *** p < 0.001 (*n* = 4).

Yeast microcapsules that contained *IL-1β* shRNA or control shRNA were respectively constructed by transforming plasmids pIN-hU6-*IL-1β* shRNA or pIN-hU6-ctrl shRNA into yeast. Based on uracil gene in shRNA plasmid (Figure S1B), yeast only containing shRNA^+uracil^ can survive in uracil selective medium (Figure 1A). Otherwise, the yeast^−uracil^ without shRNA plasmid could not survive in the uracil^−^ selective medium. Monoclonal colony PCR results showed that the constructed shRNA^+uracil^/yeast^−uracil^ microcapsules with shRNA vector present the predicted length (Figure 1B). Thus, *IL-1β* shRNA/yeast (Figure 1C) and Ctrl shRNA/yeast was successfully constructed.

LPS induced RAW264.7 macrophage was used to detect *IL-1β* shRNA/yeast function in vitro. It was observed that yeast microcapsules could be engulfed by macrophage 4 h after being added into macrophage culture medium (Figure 1D). After 24 h culture, total RNA was extracted for RT-qPCR detection. Compared to the yeast and Ctrl shRNA/yeast group, *IL-1β* and *TNF-α* expression in *IL-1β* shRNA/yeast group was down regulated (Figure 1E). The results indicated that *IL-1β* shRNA/yeast could be engulfed by macrophages and reduced the expression of inflammatory gene.

### 3.4. Yeast Microcapsule Protected shRNA from the Degradation in Gastrointestinal Tract

**Figure 2:**
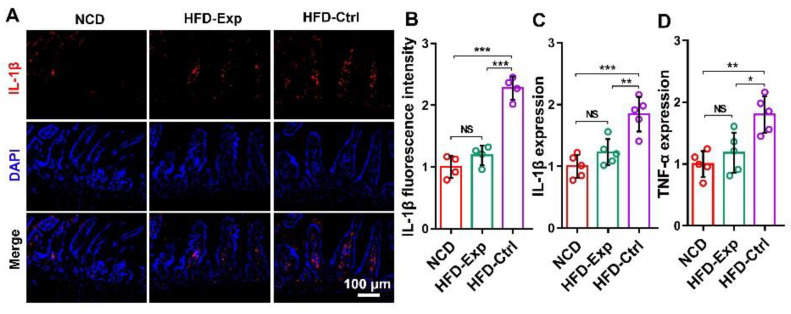
*IL-1β* shRNA/yeast inhibits IL-1β expression in small intestine and intestinal macrophages. (**A**) Immunofluorescence staining of IL-1β (red) in small intestine. DAPI nuclear staining (blue) was also conducted. (**B**) Quantification of IL-1β fluorescent signal in the small intestine (*n* = 4). (**C**,**D**) The expression of *IL-1β* and *TNF-α* in intestinal macrophage (*n* = 5). NS (no significance). * p < 0.05, ** p < 0.01, *** p < 0.001.

Small intestine tissues were collected to detect IL-1β expression on day 29. The results of immunofluorescence showed that IL-1β in HFD-Exp group was inhibited (Figure 2A). The statistics of fluorescence intensity are shown in Figure 2B. And IL-1β expression in the lung tissue (Figure S3) was similar to that in the small intestine. Macrophages, as one of the most important antigen-presenting cells, participate in the uptake and presentation of yeast microcapsule. RT-qPCR results showed that *IL-1β* and *TNF-α* expression in HFD-Exp macrophages were lower than that in the HFD-Ctrl group (Figure 2C,D). These results indicated that oral delivery of shRNA mediated by yeast microcapsules successfully passed through the gastrointestinal tract and regulated IL-1β expression in the small intestine and other organ.

### 3.5. IL-1β shRNA/Yeast Regulated Body Weight via Oral Route In Vivo

**Figure 3:**
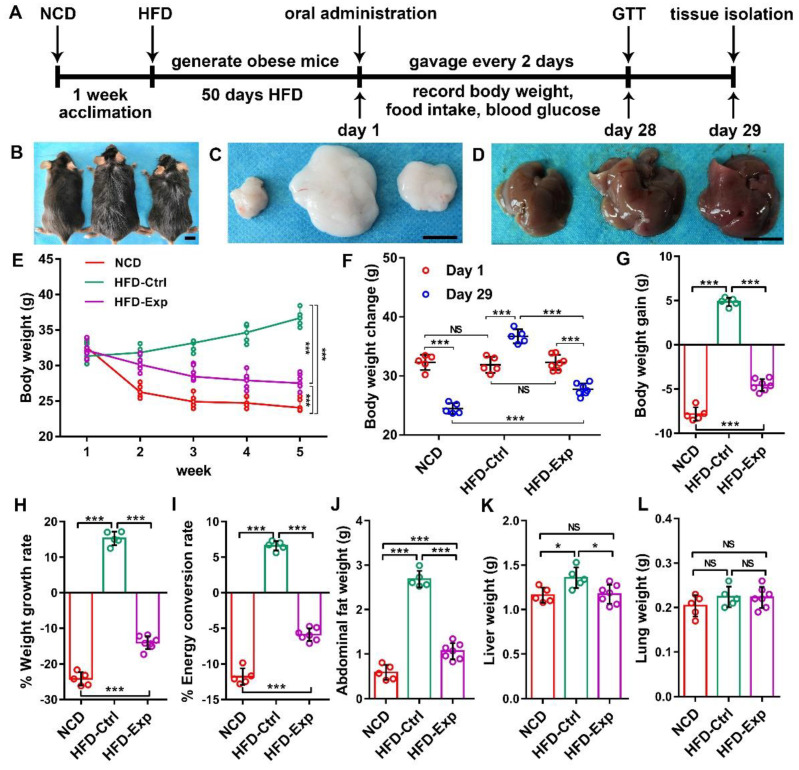
*IL-1β* shRNA/yeast regulated body weight via oral route in vivo. (**A**) Timeline of mice processing. Morphological differences of mice body type (**B**), abdominal fat (**C**) and liver (**D**) (Scale = 1 cm). From left to right, it refers to group NCD, HFD-Ctrl and HFD-Exp respectively. (**E**,**F**) Body weight changes from day 1 to day 29. Net weight gain (**G**) and net weight growth rate (**H**) of mice from day 1 to day 29. (**I**) Energy conversion rate (%). (**J**) Abdominal fat weight. (**K**) Liver weight. (**L**) Lung weight. NS (no significance). * p < 0.05, *** p < 0.001 (*n* = 5 or 7).

C57BL/6 male mice were acclimated fed with normal chow diet for one week. Then, mice were fed with high-fat diet to generate obesity model mice for 50 days. From day 1 to day 29, mice were fed with 10 mg/kg of *IL-1β* shRNA/yeast or control shRNA/yeast. The operation timeline of the entire process was shown in Figure 3A. Mouse body shape, abdominal fat and liver shape was shown in Figure 3B–D. Since obese mice in NCD group were fed with normal chow diet (low energy intake group) from day 1, the body weight decreased dramatically in the first two weeks, and then remained stable. Compared to HFD-Ctrl (high-fat diet with control shRNA/yeast), body weight in the HFD-Exp group (high-fat diet with *IL-1β* shRNA/yeast) decreased steadily (Figure 3E, Table 2 and Table S2). Compared to the body weight changes between day 1 and day 29, we found that reduced energy intake (NCD) or oral administration of *IL-1β* shRNA/yeast can both reduce body weight (Figure 3F–H). By comparing the food intake, we found that although the food collected in HFD-Exp group was higher than that in the HFD-Ctrl, the energy consumption rate was significantly lower (Figure 3I and Table 3). By comparing abdominal fat (Figure 3J), liver (Figure 3K) and lung weight (Figure 3L), we found that the main reason of weight loss in mice was the decrease of fat weight. This suggested that *IL-1β* shRNA/yeast didn’t control food intake but increased energy consumption to achieve the purpose of weight loss in obese mice. In addition, lean mass of myosin heavy chain (MHC) and myostatin (MSTN) expression (Figure S4) in muscle also proved that *IL-1β* shRNA/yeast reduced body weight by inhibiting fat content rather than muscle.

### 3.6. Histological Staining Proved the Effect of IL-1β shRNA/Yeast in Anti-Obesity

**Figure 4:**
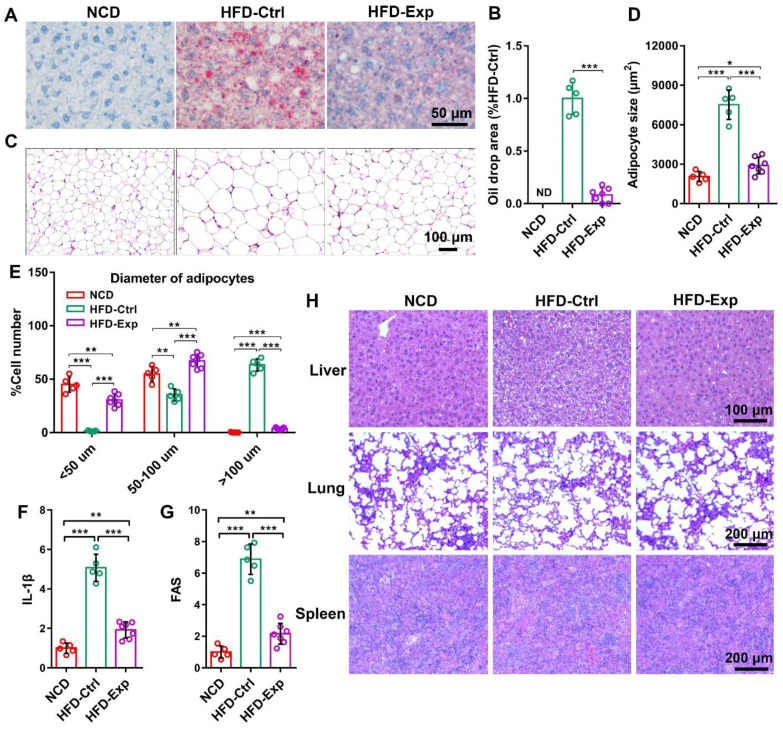
Nontoxic *IL-1β* shRNA/yeast inhibited the growth of adipocytes in liver and abdominal adipose. (**A**) Oil-red O staining of liver. (**B**) Percentage of oil drop area in liver. (**C**) H&E staining of abdominal adipose. (**D**) Adipocytes mean area (μm^2^). (**E**) Quantity statistics of adipocytes in different sizes (maximum diameter of cells, <50 μm, 50–100 μm, >100 μm). The expression of *IL-1β* (**F**) and lipid metabolism related gene FAS (**G**) in abdominal adipose. (**H**) H&E staining of liver, lung and spleen. ND (not detected). * p < 0.05, ** p < 0.01, *** p < 0.001 (*n* = 5 or 7).

Compared to HFD-Exp, liver oil-red O staining showed more fat accumulation in HFD-Ctrl group (Figure 4A,B). Abdominal fat Hematoxylin-eosin staining showed that the average size of adipocytes in HFD-Ctrl group was larger than those in the NCD and HFD-Exp group (Figure 4C). Through quantitative statistics of adipocyte size, oral gavage of *IL-1β* shRNA/yeast significantly inhibit the growth of adipocytes (Figure 4D,E). The HFD-Ctrl group had the largest number of adipocytes with a diameter greater than 100 μm (Figure 4E). And the expression of *IL-1β* and lipid metabolism related gene FAS was down regulated after *IL-1β* shRNA/yeast therapy in abdominal fat (Figure 4F,G).

### 3.7. Biological Safety of shRNA/Yeast Microcapsule In Vivo

We used H&E staining to assess the biological safety of this shRNA delivery system in vivo. Obese mice in HFD-Ctrl or HFD-Exp were respectively gaveged with 10 mg/kg of Ctrl shRNA/yeast or *IL-1β* shRNA/yeast microcapsules every two days for 29 days. The H&E staining of lung, liver and spleen showed that there was no significant difference in cell structure and tissue integrity between shRNA/yeast groups and NCD group (Figure 4H). It implied that yeast microcapsules have no toxic effect on tissues, which is consistent with our previous study [19].

### 3.8. IL-1β shRNA/Yeast Regulated Blood Glucose Concentration and Cytokines Expression in Serum

**Figure 5:**
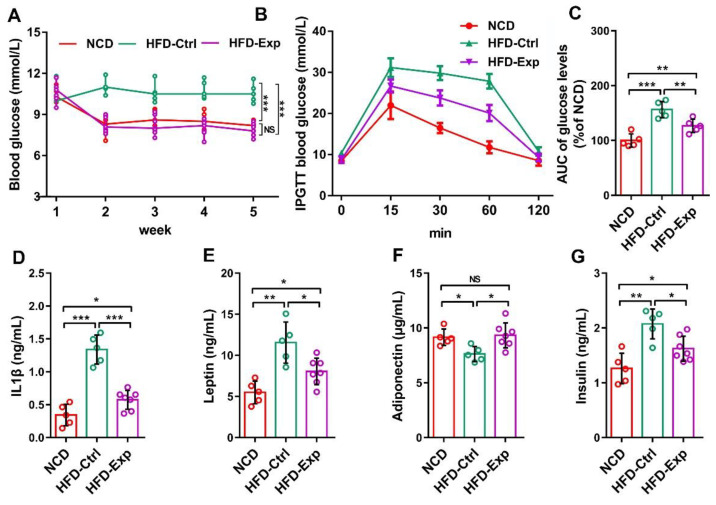
*IL-1β* shRNA/yeast regulated blood glucose concentration and cytokines expression in serum. (**A**) Changes of blood glucose concentration. (**B**) Glucose tolerance test. (**C**) Area under curve (AUC) of glucose levels after intraperitoneal injection of glucose. (**D**–**G**) The expression of IL-1β, leptin, adiponectin and insulin in the serum. NS (no significance). * p < 0.05, ** p < 0.01, *** p < 0.001 (*n* = 5 or 7).

Obese mice in HFD-Ctrl showed higher blood glucose concentrations than those in the normal chow diet group and the *IL-1β* shRNA/yeast group (Figure 5A). After mice were given an intraperitoneal glucose tolerance test, the results showed that mice in HFD-Exp could prevent acute glucose elevation compared to HFD-Ctrl group (Figure 5B). T’he area under curve of blood glucose was quantified and showed in Figure 5C.

ELISA was used to detect the expression of IL-1β, leptin, adiponectin and insulin in serum. The expression of IL-1β, insulin and leptin in HFD-Ctrl mice was higher than those in lean mice (Figure 5D,E,G). In contrast, the expression of adiponectin in obese mice was lower than that in lean mice (Figure 5F). This suggested that oral administration of shRNA/yeast could systematically regulate the expression of obesity related proteins in blood.

### 3.9. Weight Loss Alleviates Articular Cartilage Injury

Previous studies showed that obesity could aggravate knee joint involvement, which leads to articular cartilage damage [22,23,24,25]. In this study, Safranin O/Fast Green and immunohistochemical staining were used to explore the function of *IL-1β* shRNA/yeast on weight loss and joint injury from the perspective of pathology. Safranin O/Fast Green results showed that the articular cartilage in NCD, HFD-Ctrl, and HFD-Exp groups accompanied with different degrees of injury. This suggested that previous obesity did cause articular cartilage injury. Compared with NCD group, mice in HFD-Ctrl group with high-fat diet led to further obesity and joint injury (Figure S5). On the contrary, as the result of weight loss by *IL-1β* shRNA/yeast, mice joint injury in HFD-Exp group was improved. This was consistent with our previous results that regulating *IL-1β* expression can improve the symptoms of osteoarthritis [18]. Among the three groups, articular cartilage in the HFD-Exp showed the best proteoglycan staining and intact surface (Figure S5A). And immunohistochemistry results revealed that the expression of cartilage injury related protein MMP13 and IL-1β in the NCD and HFD-Ctrl groups was higher than the HFD-Exp group (Figure S5A). High expression of MMP13 and IL-1β increased cartilage injury formation. Articular cartilage damage score was individually evaluated by three professional persons using OARSI standards. The score of MFC (medial femoral condyle) and MTP (medial tibial plateau) in HFD-Ctrl group were much higher than the HFD-Exp group (Figure S5B). Compared to HFD-Ctrl, *IL-1β*, *MMP13* and *Col X* (hypertrophic marker col10a1) was down regulated while anabolic cartilage matrix marker *Col II* (col2a1) level was up regulated in the cartilage joint of NCD and HFD-Exp groups (Figure S5C). These results suggested that both of diet control and gene regulation by *IL-1β* shRNA could reduce body weight and further alleviate the articular cartilage degeneration caused by obesity.

## 4. Discussion

Obesity is a chronic systemic inflammation, which can induce macrophages to produce IL-1β and increase the circulating IL-1β concentration. Studies have shown that inhibiting IL-1β can improve β-cell function and glycemic control in obese mice [6,7]. IL-1β is characterized as a new fat secreted adipokine which could elicit beta-cell demise [26] and stimulate glucose uptake in adipose tissue [6]. In this study, we developed a novel weight loss strategy through yeast microcapsule mediated oral delivery of *IL-1β* shRNA to regulate the expression of obesity-related gene and inflammatory response.

Yeast microcapsule as oral delivery vehicle has the following advantages: (1) As baker’s or brewer’s yeast, yeast is safe and widely used in wine, beer making, and food [10]. Unlike bacteria, food grade yeast is safe and often used in vaccine development [27,28]; (2) yeast can effectively resists the digestion and degradation of the gastrointestinal tract and be used in nano-materials, gene or protein drugs delivery by oral method [11,12,13,14,15]. And we proved that yeast microcapsule can be used for delivery of shRNA or miRNA for the treatment of osteoarthritis via the oral route in vivo [18,19]; (3) as the main component of the yeast wall [29], β-glucan could be specifically recognized by dectin-1 receptor on the surface of macrophage [30]. Based on its targeting to macrophages, it plays an important role in immunoregulation. Studies also showed that yeast can be transferred from the gut and infiltrated into other tissues after being engulfed by intestinal macrophages [14,15,16,17]. We also demonstrated that yeast microcapsule mediated oral miRNA or shRNA delivery could regulate macrophage related inflammatory response for the treatment of post-traumatic osteoarthritis [18,19].

Depending on mouse *IL-1β* mRNA sequence, we designed three different shRNA targeted sequences based on bioinformatic analysis. The shRNA sequences were, respectively, cloned into pIN-hU6 plasmid [18]. Our in vitro study showed that *IL-1β* shRNA can not only inhibit the expression of IL-1β in macrophages, but also inhibit the expression of lipid in adipocytes. Based on the design of pIN-hU6-shRNA plasmid, shRNA^+uracil^ plasmid was transfected into uracil deficient yeast. Then shRNA yeast was screened in the uracil deficient medium. The function of *IL-1β* shRNA/yeast was detected with LPS induced RAW264.7 macrophage in vitro. *IL-1β* shRNA/yeast could not only be specifically recognized and phagocytized by macrophages, but also inhibited IL-1β expression in macrophages. 

As the first organ involved in shRNA/yeast uptake, the small intestine was collected to detect IL-1β expression after *IL-1β* shRNA/yeast treatment. Previous studies showed that intestinal macrophages participated in the uptake and presentation of yeast [14,15]. Down-regulation of IL-1β demonstrated that yeast microcapsules successfully delivered shRNA to the gut without being digested by the gastrointestinal tract via the oral route. These features provide that *IL-1β* shRNA/yeast can be used in obesity therapy. Study also showed that yeast microcapsules mediated drug delivery can be used for atherosclerosis therapy through oral method [14]. The phagocytosed yeast accompanied with macrophages could traffic away from the gut and infiltrate to other tissues [14,15,16,17]. Previously, studies also proved that yeast-mediated oral miRNA or shRNA delivery can modulate macrophage related inflammatory response in the treatment of osteoarthritis [18,19]. Finally, yeast-macrophages participate in immune regulation or gene therapy in inflammatory tissues [12,18].

Diet-induced obesity is a state of pre-diabetes, manifested by lipid accumulation and low-grade inflammation [20]. Therefore, lipid metabolism and inflammatory proteins can be used as reference factors for evaluating the effect of obesity treatment. In this project, body weight and fat weight in obese mice were significantly reduced after *IL-1β* shRNA/yeast treatment. By comparing multiple tissues or organs, we found that the decline of the abdominal fat was the main factor in weight loss. The expression of myosin heavy chain and myostatin in muscle also proved that *IL-1β* shRNA/yeast had no effect on muscle development. Compared to the HFD-Ctrl group, mice in HFD-Exp showed higher food intake but lower energy conversion rate. This indicated that the role of *IL-1β* shRNA/yeast was not to suppress appetite, but to achieve weight by promoting fat metabolism. In order to further prove this conclusion, we analyzed the abdominal fat. Abdominal fat H&E staining showed that the size of adipocytes in HFD-Exp group was much smaller than those in the HFD-Ctrl group. The growth of adipocytes was significantly inhibited by *IL-1β* shRNA/yeast. And the expression of *IL-1β* and lipid metabolism related gene FAS in abdominal fat was down regulated after *IL-1β* shRNA therapy. Compared with NCD group, we found that although *IL-1β* shRNA/yeast can reduce weight gain by increasing fat metabolism, reducing energy intake has a better effect on weight loss. It is easy to understand that reducing energy intake by diet control is more direct and effective than increasing energy metabolism by drug treatment.

Leptin is related to the body’s energy balance, and the level of leptin in obese mice is significantly increased [31]. Leptin is a satiety hormone, which can promote the reduction in food intake. However, sustained high levels can also promote leptin resistance. Since leptin is secreted by adipose, these changes may be caused by the changes in fat mass [32,33]. Adiponectin is directly related to insulin sensitivity [34]. It was found that adiponectin in the blood circulatory system was slightly higher than that in NCD group obese mice, while leptin and insulin was inhibited after *IL-1β* shRNA/yeast treatment. It can effectively maintain the change of blood glucose concentration. Therefore, *IL-1β* shRNA/yeast can promote fat metabolism and reduce body weight without controlling food intake. It is good news to obese patients who need to lose weight but cannot control their diet.

Evidence showed that chronic joint disease, osteoarthritis (OA), involves in a state of inflammatory status and is associated with obesity [22,23]. Obesity is related to OA due to increased body weight, metabolic factors [24], and systemic inflammatory effects [25]. Such OA-causing effect involves many genes, such as *IL-1β* [35], one of the key genes in OA pathogenesis. Studies have shown that *IL-1β* can accelerate matrix metalloproteinases (MMPs) expression and promote the decomposition of collagen and proteoglycan in cartilage [36,37]. Moreover, we have previously confirmed that inhibition of *IL-1β* can effectively improve OA symptoms [18]. In this study, mice knee joints were collected to assess whether the body weight loss alleviated articular cartilage injury.

Inhibition of IL-1β lead to down regulation of MMP13 and Col X but up regulation of anabolic marker Col II. Histological staining results suggested that *IL-1β* shRNA/yeast can improve joint damage caused by obesity during weight loss. Probably because this study was completed in a relatively short period of time, obesity caused moderate damage to articular cartilage, which is not as serious as in patients with advanced OA. However, there was significant difference in the degree of joint damage between HFD-Ctrl and HFD-Exp. These results indicated that *IL-1β* shRNA/yeast could reduce body weight and further alleviate the articular cartilage degeneration caused by obesity.

## 5. Conclusions

In conclusion, this study showed that *IL-1β* shRNA/yeast can effectively reduce body weight and further alleviate the articular cartilage degeneration caused by obesity. Compared with parenteral administration, yeast microcapsule-mediated oral delivery of gene drugs for obesity treatment not only has high patient compliance, but also is non-invasive and requires less patient supervision. This kind of non-diet-controlled weight loss strategy brings good news to obese patients who need to lose weight but cannot control their diet.

## Figures and Tables

**Table 1:** Information of primers sequences.

Primer Name	Sequence (5′ to 3′)
shDec-F	AGAATCGTTGCCTGCACATC
shDec-R	GAGATAGCAAGGTATTCAG
IL-1β-F	AACCTGCTGGTGTGTGACGTTC
IL-1β-R	CAGCACGAGGCTTTTTTGTTGT
18S-F	CGGCTACCACATCCAAGGAA
18S-R	GCTGGAATTACCGCGGCT
FAS-F	CTGAGATCCCAGCACTTCTTGA
FAS-R	GCCTCCGAAGCCAAATGAG
TNF-α-F	ACCCTCACACTCAGATCATCTTC
TNF-α-R	TGGTGGTTTGCTACGACGT
MHC-F	GGAAGACCGCAAGAATGTTCTC
MHC-R	CAGGTTGACATTGGATTGTTCCT
MSTN-F	CAAAACACGAGGTACTCCAGAATAG
MSTN-R	CCGTGGTAGCGTGATAATCGT
Col II-F	AGGACCTGGTAGTGCGAGTGAC
Col II-R	CTGGGCGATAGTGGAATACAACT
Col X-F	GATGCCGCTTGTCAGTGCT
Col X-R	CCAGATCTTGGGTCGTAATGC
MMP13-F	GCCCCTTCCCTATGGTGATG
MMP13-R	TTGCATTTCTCGGAGCCTGT

**Table 2:** Mice body weight.

	Week 1	Week 2	Week 3	Week 4	Week 5
NCD (g)	32.30 ± 1.29	26.39 ± 0.93	25.02 ± 0.94	24.87 ± 0.77	24.47 ± 0.91
HFD-Ctrl (g)	31.87 ± 1.36	31.86 ± 0.92	32.80 ± 0.76	34.54 ± 1.24	36.72 ± 1.18
HFD-Exp (g)	32.82 ± 1.28	30.37 ± 1.09	28.86 ± 0.98	27.83 ± 1.10	27.73 ± 1.01

**Table 3:** Food intake and energy conversion efficiency from day 1 to day 29.

	Total Intake (g)	Average Net Intake Per Mouse (g)	Net Gain Per Mouse (g)	Energy Conversion Rate (%)
NCD (*n* = 5)	332.12	66.42	−7.82 ± 0.77	−11.78 ± 1.16
HFD-Ctrl (*n* = 5)	367.21	73.44	4.85 ± 0.48	6.60 ± 0.65
HFD-Exp (*n* = 7)	538.83	76.98	−4.55 ± 0.67	−5.91 ± 0.87

## Data Availability

Data is contained within the article or supplementary material.

## Supplementary Materials

The following are available online at www.mdpi.com/article/10.3390/pharmaceutics13101536/s1. Figure S1: Information of shRNA expression vector and efficiency detection of *IL-1β* shRNA in 293T cells in vitro; Figure S2: *IL-1β* shRNA increased lipid metabolism in adipocytes; Figure S3: Oral administration of *IL-1β* shRNA/yeast inhibits lung IL-1β expression; Figure S4: *IL-1β* shRNA/yeast has no effect on myosin heavy chain and myostatin expression in muscle; Figure S5: Inhibition of weight gain alleviated articular cartilage injury; Table S1: The sequences of *IL-1β* shRNA and its target sites; Table S2: Average net weight gain per week.
