# Supplementary material for: Oral Gene Therapy of HFD-Obesity via Nonpathogenic Yeast Microcapsules Mediated shRNA Delivery
Source: Pharmaceutics. 2021 Sep 22;13(10):1536. doi: 10.3390/pharmaceutics13101536 (PMC8539367; doi:10.3390/pharmaceutics13101536)
Supplement: Supplementary file 1 [file pharmaceutics-13-01536-s001.zip › pharmaceutics-1338255-supplementary.pdf]

Supplementary Materials: Oral Gene Therapy of HFD-obesity *via* Nonpathogenic Yeast Microcapsules Mediated shRNA Delivery

Li Zhang, Wan Zhang, Hang Peng, Yankun Li, Tongtong Leng, Chenxi Xie and Long Zhang

Table S1. The sequences of *IL-1β* shRNA and its target sites.

shRNA Name	Sequence (5' to 3')	Target Site (5' to 3')
IL-1β shRNA 1	**GACAGAATATCAACCAACA**ATA*GTGAAGCCACAGATG*TAT**TGTTGGTTGATATTCTGT**	ATGGACAGAATATCAACCA
IL-1β shRNA 2	**CACATTCTGTTCAAAGAGA**GTA*GTGAAGCCACAGATG*TAC**TCTCTTTGAACAGAATGTG**	TGGCACATTCTGTTCAAAG
IL-1β shRNA 3	**CATTGAAGCTGAGAATAAA**TTA*GTGAAGCCACAGATG*TAA**TTTATTCTCAGCTTCAATG**	TTTCATTGAAGCTGAGAAT

Notes: **Bold** sequence refers to the functional sequence of shRNA and *italic* sequence refers to the loop structure of shRNA.

Table S2. Average net weight gain per week (g).

	week 1	week 2	week 3	week 4	week 5
NCD (*n* = 5)	0	-5.90	-1.38	-0.14	-0.40
HFD-Ctrl (*n* = 5)	0	-0.02	0.94	1.74	2.19
HFD-Exp (*n* = 7)	0	-1.91	-1.51	-1.03	-0.11

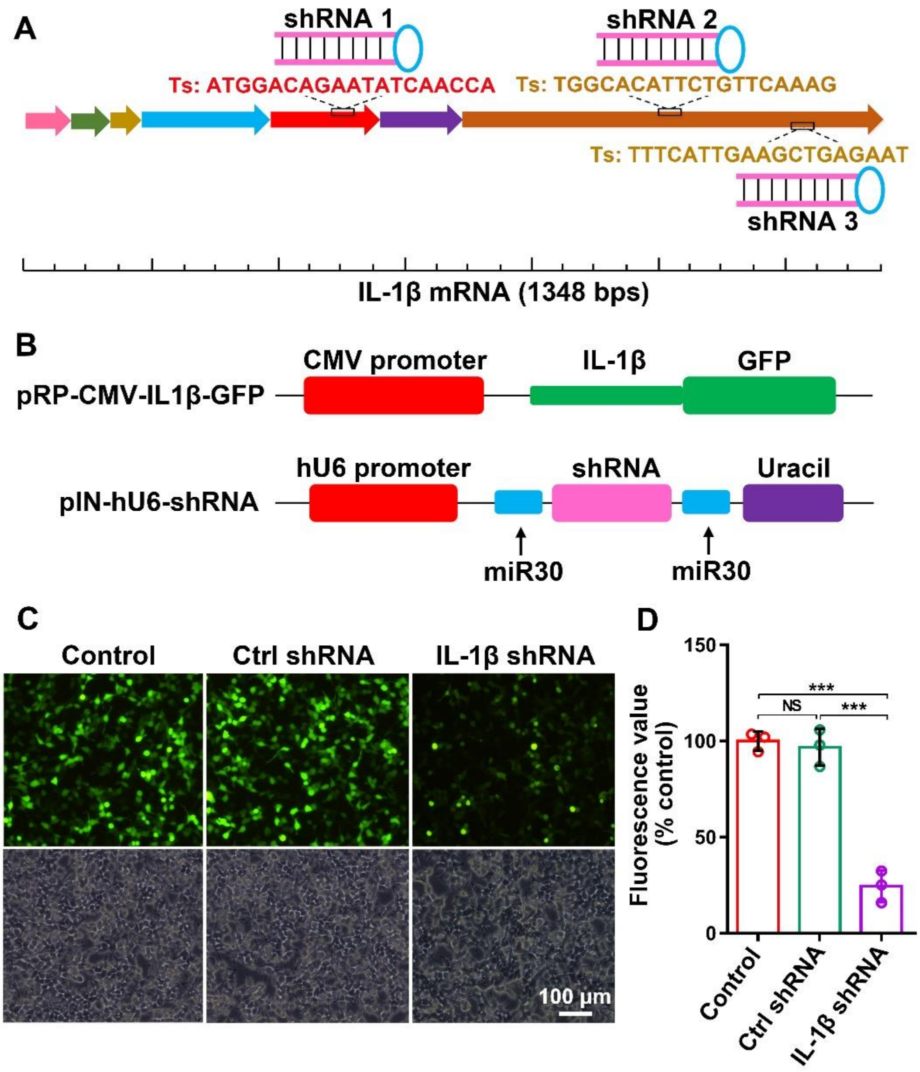

Figure S1. Information of shRNA expression vector and efficiency detection of *IL-1β* shRNA in 293T cells in vitro. **(A)** The shRNA target sites and target sequences (Ts) on the *IL-1β* mRNA. **(B)** The structure of pRP-CMV-IL1β-GFP (pRP) and pIN-hU6-shRNA plasmid. **(C)** Functional detection of *IL-1β* shRNA in 293T cells ($n = 3$). **(D)** Fluorescence intensity. Data was expressed as mean ± SD. NS (no significance). *** $P < 0.001$.

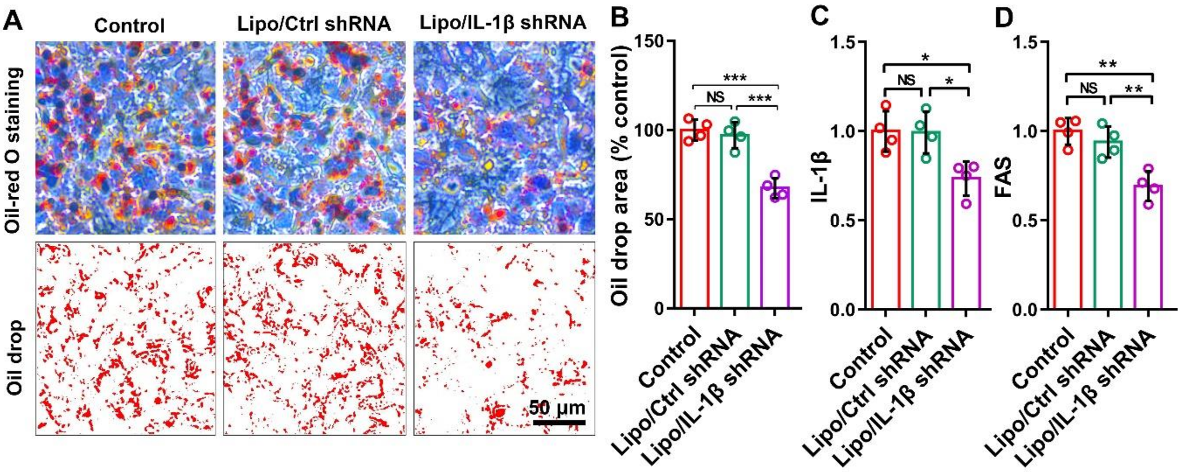

Figure S2. *IL-1β* shRNA increased lipid metabolism in adipocytes. The function of *IL-1β* shRNA on inhibition of fat expression was evaluated by Oil-Red O Staining and RT-qPCR. **(A)** Oil-Red O Staining and oil drop area. **(B)** Percentage of oil drop area. **(C–D)** Expression of fat related genes *IL-1β* and *FAS*. NS (no significance). * $P< 0.05$, ** $P< 0.01$, *** $P< 0.001$ ($n = 4$).

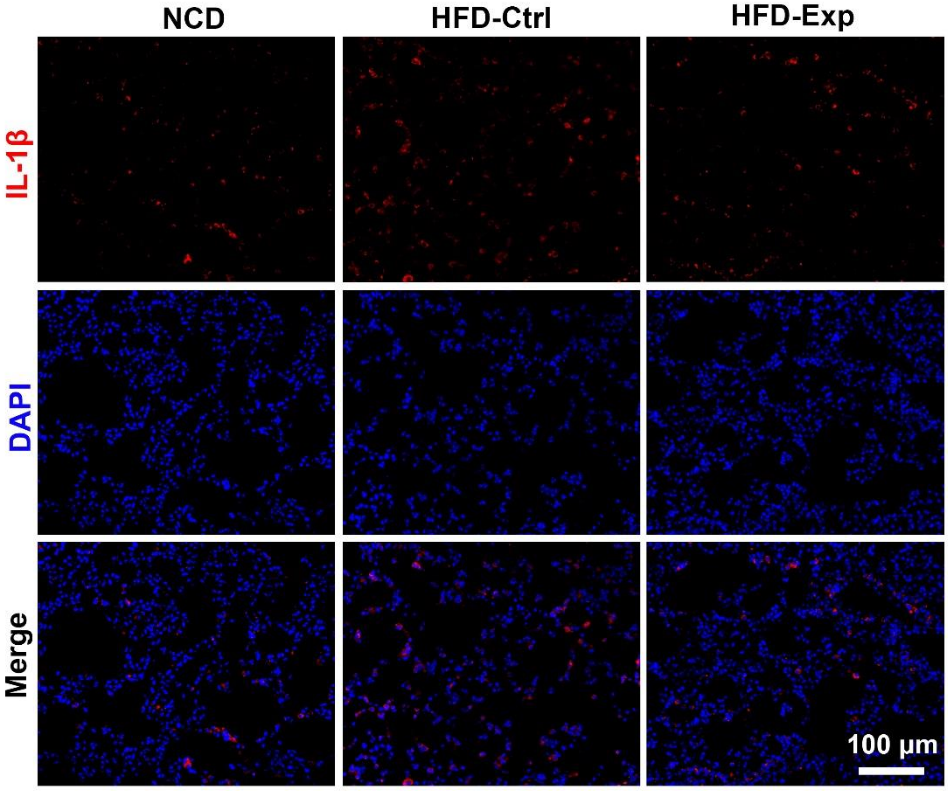

Figure S3. Oral administration of IL-1 β shRNA/yeast inhibits lung IL-1β expression. Immunofluorescence staining of IL-1β (red) in the lung tissue. DAPI nuclear staining (blue) was also conducted ($n = 4$).

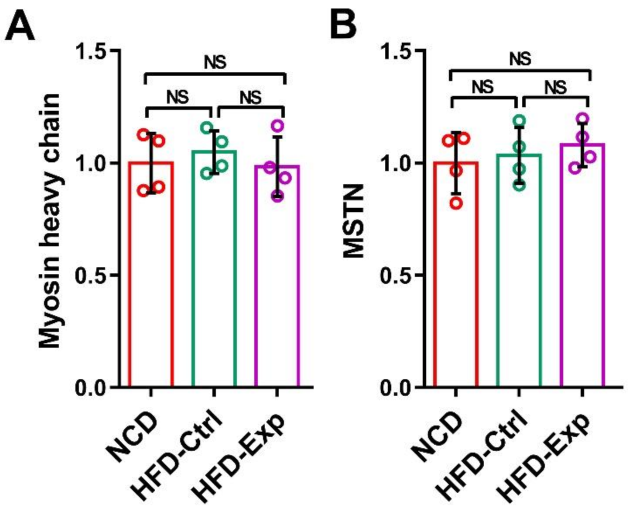

Figure S4. *IL-1β* shRNA/yeast has no effect on myosin heavy chain and myostatin (MSTN) expression in muscle. The expression of MHC (A) and MSTN (B) in muscle. Data was expressed as mean ± SD. NS (no significance) (n = 4).

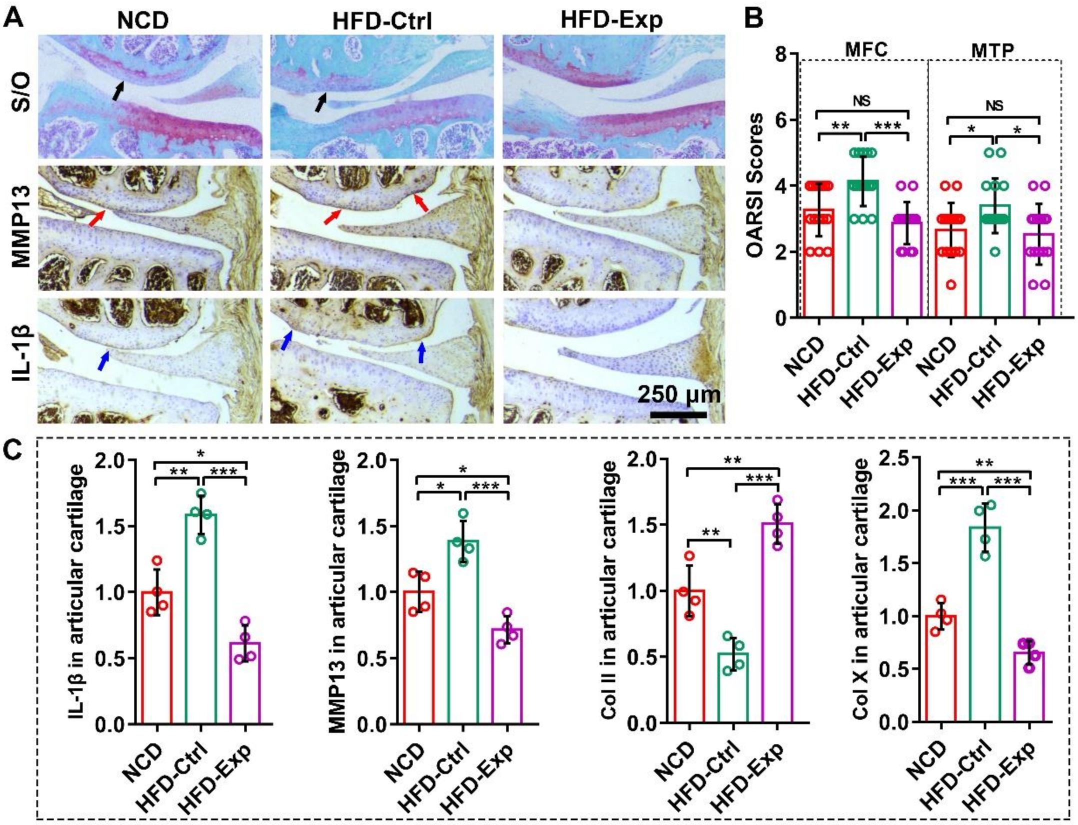

Figure S5. Inhibition of weight gain alleviated articular cartilage injury. **(A)** Safranin O/Fast Green and immunohistochemistry staining of knee joint. **(B)** Articular cartilage damage scores of the medial tibial plateau (MTP) and medial femoral condyle (MFC) were assessed using OARSI criteria (n = 15). **(C)** Related gene expression of cartilage formation or degradation in the knee joint (n = 4). NS (no significance). * P<0.05, ** P<0.01, *** P<0.001.
